# Prevalence of malnutrition and associated factors in Chinese children and adolescents aged 3–14 years using machine learning algorithms

**DOI:** 10.7189/jogh.15.04204

**Published:** 2025-07-21

**Authors:** Fangjieyi Zheng, Kening Chen, Xiaoqian Zhang, Qiong Wang, Zhixin Zhang, Wenquan Niu

**Affiliations:** 1 Centre for Evidence-Based Medicine, Capital Institute of Paediatrics, Beijing, People’s Republic of China; 2 China-Japan Friendship Hospital, Chinese Academy of Medical Sciences & Peking Union Medical College, Beijing, People’s Republic of China; 3 Graduate School, Beijing University of Chinese Medicine, Beijing, People’s Republic of China; 4 Department of Paediatrics, China-Japan Friendship Hospital, Beijing, People’s Republic of China; 5 International Medical Services, China-Japan Friendship Hospital, Beijing, People’s Republic of China

## Abstract

**Background:**

Child malnutrition represents a critical global public health issue and it is characterised by high prevalence and severe long-term consequences for growth and development. A better understanding of its contributory factors is essential to inform the design of targeted prevention strategies and evidence-based interventions. We aimed to estimate the prevalence of malnutrition in children and adolescents aged 3–14 years, and further to identify promising factors associated with child malnutrition using machine learning algorithms.

**Methods:**

Thirty kindergartens and 26 schools were randomly selected from Beijing and Tangshan. Child malnutrition was defined according to WHO standards. Factors for child malnutrition were selected by Logistic regression and three ensemble learning algorithms. An open-access web platform was developed to facilitate calculating probabilities of child malnutrition.

**Results:**

Total 18 503 children and adolescents were surveyed, and 10.93% (n = 2022) of them were found to be malnourished. Random forest emerged as the best model, as it carried the highest area under the receiver operating characteristic curve (AUROC) at 0.929. Under the implementation of random forest, top eight factors that formed the optimal set for child malnutrition prediction were identified, including age, frequency of fast food intake, frequency of late-night snacking, family history of diabetes, duration of breastfeeding, sedentary time, and parental body mass index. Further Logistic regression analyses confirmed the predictive significance of these individual factors.

**Conclusions:**

We have identified eight contributory factors for malnutrition in 3–14-year-old children and adolescents in Beijing and Tangshan, with their prediction performance optimal under random forest. More studies among independent populations are warranted to validate our findings.

Child malnutrition, which encompasses stunting, wasting, and underweight conditions, is characterised by inadequate or unbalanced consumption of protein, energy, and essential nutrients [1]. As a global public health concern, child malnutrition demands greater awareness and effective intervention. Latest updates from the World Health Organization (WHO) in 2022 show that 148.1 million children under five years old are affected by stunting, and 45.0 million by wasting [2]. In China, the prevalence of underweight and stunting among children under five was estimated at 3.6% and 9.9% [3], respectively, while the prevalence of malnutrition among 7–18-year-old children is 8.6% [4]. The underlying causes of child malnutrition are complex, ranging from short-term effects such as impaired immune system and delayed physical growth and development to long-term effects such as cognitive impairment and delayed mental development, and even an increased risk of mortality [5,6]. Additionally, child malnutrition can impede economic growth and national development [7]. Above lines of evidence underscores the importance of identifying factors responsible for child malnutrition and implements effective prevention strategies to curb this global issue.

The development of malnutrition is a highly intricate process influenced by both inherited and environmental factors. These factors operate independently, with each inherited predisposition and environmental exposure playing a dominant role, and interactively, through their complex interplay, ultimately shaping child’s nutritional status. Prior studies have corroborated the contribution of socioeconomic and environmental factors to child malnutrition [8,9]. Recently, the impact of dietary factors on child malnutrition has been a subject of active discussion, especially regarding the lack of dietary diversity among children [8],and the excessive intake of red meat, oil, and salt. A rising concern is nutritional imbalance [10–12]. Also, there is evidence for the influence of parental lifestyle, education attainment, and family income on child weight status [13]. In the existing literature, the majority of observational studies have focused on one or a few of factors mentioned above, overlooking comprehensive evaluation of a wide panel of contributing factors.

To fill this gap in knowledge and yield more information, we, in 18 503 children and adolescents aged 3–14 years from Beijing and Tangshan, aimed to estimate the prevalence of malnutrition and determine a minimum set of culprit factors that can capture the risk of child malnutrition by employing multiple machine learning algorithms, with the ultimate goal of informing targeted interventions to improve child health outcomes.

## METHODS

The conduct of this cross-sectional survey followed the checklist of STROBE (STrengthening the Reporting of OBservational studies in Epidemiology) statement (Table S1 in the **Online Supplementary Document**).

### Study design and participants

During the period between September 2020 and January 2022, we executed two cross-sectional surveys. The first was conducted in Beijing and Tangshan, a city located within Hebei province from September to December of 2020. The second survey was conducted in the Pinggu district, Beijing in January 2022.

The selection process of study participants has been detailed priorly [14,15]. For the first survey, the cohort comprised preschool-aged children enrolled in junior, middle, and senior kindergarten classes. Using stratified cluster sampling techniques, four districts from Beijing and two districts from Tangshan were randomly selected. Five kindergartens were chosen from each selected district, yielding a total of 30 kindergartens. For the second survey, participants comprised primary and junior high school students. In the Pinggu district, Beijing, 26 schools were randomly selected, encompassing eight primary schools and 18 junior high schools. Data were collected from 3–14-year-old children attending these 30 kindergartens and 26 schools in Beijing and Tangshan.

While the study employed a stratified cluster sampling approach, it is recognised that the manuscript did not clearly outline statistical calculations like effect size, power analysis, or confidence interval-based formulas, to determine the sample size. In cross-sectional studies, sample size calculations typically rely on expected prevalence, allowable error margins, and confidence levels. To illustrate this point, we a hypothetical scenario was considered. Given a malnutrition prevalence of 10.93% (as observed), a 95% confidence level (Z = 1.96), and a 0.5% margin of error, the theoretical sample size would be approximately 1500. Nevertheless, the actual final sample size of 18 503 is substantially larger than this estimate. This large sample size ensures high prevision and robustness for subgroup analyses and machine-learning modeling.

### Data collection and variable definition

Baseline information including children’s demographic characteristics (age, sex, nationality, date of birth, height, weight, food and drug allergy), fetal and neonatal information (birth body length, gestational age, delivery mode, pregnancy order, delivery order, assisted reproduction, twin birth, infancy feeding, breastfeeding duration, and time of adding solid food), sitting time, screen time, outdoor activity time, sleep duration, fall asleep time, eating speed, number of dental caries, and weekly intake frequencies of sweet food, night meals, and fast food, parental age, parental weight, parental height, parental education, and family income was gleaned via standardised questionnaires. Body mass index (BMI) was calculated as weight divided by height squared (kg/m^2^).

Covariates included age, sex, and nationality, which are unchangeable, and the other factors were treated as independent variables of interest. Both covariates and variables were incorporated into the machine learning models.

### Quality control

Before distributing our self-designed questionnaires, health care physicians and responsible teachers, selected from selected schools and kindergartens for this survey, received comprehensive training to ensure familiarity with the survey procedures and questionnaire items. During survey process, trained professionals assisted parents or guardians of participating children in accurately completing the questionnaires. Collected data were exported from the Wenjuanxing (a Chinese online survey platform) to Microsoft Office Excel spreadsheets, where it underwent rigorous scrutiny by our well-trained staff.

In cases where the questionnaires contained noticeably anomalous information, health care physicians and responsible teachers were tasked with reaching out to the parents or guardians of the concerned children to provide additional details or confirm information accuracy. During the survey, health care physicians and teachers-in-charge can help parents or guardians of participating children to fill out questionnaires. Additionally, health care physicians precisely measured participants’ body weight (to the nearest 0.1kg) and height (to the nearest 0.1cm).

### Definition of malnutrition

The outcome of this study was child malnutrition, which comprises stunting, underweight, and wasting. This study strictly followed the WHO guidelines to define malnutrition in children. Precisely, the 2006 WHO Child Growth Standard [16] outlined metrics for identifying stunting, underweight, and wasting in children aged 0–5 years, and the 2007 WHO growth reference for school-aged children and adolescents [17] detailed standards for stunting and wasting in children aged 5–19 years.

The Z-score, calculated as the deviation of a child’s value from the median of the reference population divided by the standard deviation of the reference population, was utilised to assess nutritional status. Per WHO’s criteria, stunting, underweight, and wasting were defined for two age-specific groups:

i) for children aged 36–60 months, stunting was defined as a height-for-age Z-score (HAZ) less than −2 standard deviations (SDs), underweight as a weight-for-age Z-score (WAZ) below −2 SDs, and wasting as a weight-for-height Z-score (WHZ) less than −2 SDs;

ii) for children aged 61–180 months, stunting was defined as a HAZ below −2 SDs, accompanied by a BMI-for-age Z-score (BMIZ) less than −2 SDs.

### Statistical analyses

Statistical analyses were performed using the R coding platform version 4.3.2 (R Foundation for Statistical Computing, Vienna, Austria). Variables with over 30% missing data were excluded. Missing data were imputed via the MICE package (multivariate imputation by chained equations). Propensity score matching (PSM) was implemented to balance sex distributions between malnourished and normally nutritional children/adolescents. To ensure analytical robustness, data were randomly divided into two data sets: a training set comprising 70% of the data and a testing set containing the remaining 30% for cross-validation. For comparisons between groups, statistical tests were selected based on the nature of the variables. Specifically, the *t* test was applied to normally distributed continuous variables, expressed as mean (SD); the rank sum test was used for skewed continuous variables, expressed as median (interquartile range); and the χ2 test was employed for categorical variables, presented as count (percentage).

Logistic regression and three ensemble learning algorithms – decision tree, random forest, and gradient boosting machine (GBM) – were utilised to identify contributing factors related to malnutrition. The optimal algorithm was identified through a comprehensive assessment of performance metrics such as accuracy, the Brier score, and area under the receiver operating characteristic (AUROC) curves. Additional metrics included mean absolute error (MAE), mean squared error (MSE), the area over the regression error characteristic curve (REC), and the area over the regression receiver operating characteristic curve (RROC). With the optimal algorithm, the significance of the investigated factors was evaluated and ranked from the highest to lowest in predicting malnutrition. Subsequently, the optimal number of top factors was ascertained via cumulative performance evaluation, including AUROC, accuracy, and precision. Moreover, to enhance the interpretability of the factors identified by the optimal algorithm, logistic regression models were conducted for underweight, overweight, and obesity, presenting effect size estimates as odds ratios (OR) with 95% confidence intervals (95% CI).

A user-friendly Shiny web application [18] was developed to estimate the probability of child malnutrition. Data analysis was performed between March and July 2024.

### Ethical considerations

This study was approved by the Ethics Committee of China-Japan Friendship Hospital (approval No. 2018-93-K67) and Beijing University of Chinese Medicine (approval No. 2022BZYLL0906). The study conducted in two phases strictly adhered to local laws and institutional protocols. Prior to participation, written informed consent was obtained from the parents or guardians of all children and adolescents. To ensure confidentiality, all data were anonymised and assigned unique identifiers for tracking purposes.

## RESULTS

### Baseline characteristics

**Table 1** presented baseline characteristics of 18 503 children and adolescents, and 2022 of them were identified as malnourished, reflecting a prevalence of malnutrition at 10.93%.

**Table 1:** Baseline characteristics of children by the presence of malnutrition*

Characteristic	Children without malnutrition (n = 16 481)	Children with malnutrition (n = 2022)	*P*-value
Demographic characteristics			
*Age in years, median (IQR)*	7.91 (4.91–11.17)	5.50 (4.32–7.58)	<0.001
*Boys, n (%)*	8486 (51.49)	1036 (51.24)	0.830
Foetal and early life factors			
*Gestational age (weeks), median (IQR)*	39.00 (38.00–40.00)	39.00 (38.00–40.00)	0.490
Full-term birth, n (%)			0.384
*Preterm delivery*	1548 (9.39)	200 (9.89)	
*Normal*	14 446 (87.65)	1753 (86.70)	
*Post-term pregnancy*	487 (2.95)	69 (3.41)	
Delivery mode, n (%)			0.081
*Vaginal delivery*	8087 (49.07)	1043 (51.58)	
*Caesarean section*	8356 (50.70)	973 (48.12)	
*Forceps delivery*	38 (0.23)	6 (0.30)	
*Pregnancy order, median (IQR)*	1.00 (1.00–2.00)	2.00 (1.00–2.00)	<0.001
*Delivery order, median (IQR)*	1.00 (1.00–2.00)	1.00 (1.00–2.00)	<0.001
*Twin birth, n (%)*	402 (2.44)	43 (2.13)	0.387
*Birth length (cm), median (IQR)*	50 (50–52)	50 (50–52)	0.287
*Birth weight (kg), median (IQR)*	3.40 (3.00–3.60)	3.30 (3.00–3.55)	<0.001
Infancy feeding, n (%)			0.069
*Pure breastfeeding*	9307 (56.47)	1194 (59.05)	
*Partial breastfeeding*	5688 (34.51)	665 (32.89)	
*Non-breastfeeding*	1486 (9.02)	163 (8.06)	
Breastfeeding duration, n (%)			<0.001
*<6 mo*	5063 (30.72)	410 (20.28)	
*6–24 mo*	9918 (60.18)	1337 (66.12)	
*≥24 mo*	1500 (9.10)	275 (13.60)	
Time to introduce solid food, n (%)			0.001
*<6 mo*	2917 (31.45)	351 (33.69)	
*6–9 mo*	4840 (52.18)	485 (46.55)	
*≥9 mo*	1518 (16.37)	206 (19.77)	
Lifestyle-related factors			
*Sedentary time (hours per day), median (IQR)*	4.00 (2.00–6.29)	2.57 (1.29–4.86)	<0.001
*Screen time (hours per day), median (IQR)*	1.14 (0.64–1.57)	1.00 (0.64–1.57)	<0.001
*Outdoor activities (hours per day), median (IQR)*	1.28 (1–2)	1.29 (1–2.28)	<0.001
*Bedtime (o’clock PM), median (IQR)*	9.50 (9.00–10.00)	9.00 (9.00–10.00)	<0.001
*Eating speed (minutes per meal), median (IQR)*	16.67 (13.33–21.67)	18.33 (15–25)	<0.001
Fast food intake frequency, n (%)			<0.001
*Every day*	4362 (26.47)	237 (11.72)	
*3–5 times weekly*	4801 (29.13)	237 (11.72)	
*1–2 times weekly*	2694 (16.35)	536 (26.51)	
*None or once in a while*	4624 (28.06)	1012 (50.05)	
Sweet food intake frequency, n (%)			<0.001
*Every day*	2348 (14.25)	202 (9.99)	
*3–5 times weekly*	7314 (44.38)	627 (31.01)	
*1–2 times weekly*	5356 (32.50)	957 (47.33)	
*None or once in a while*	1463 (8.88)	236 (11.67)	
Night meal intake frequency, n (%)			<0.001
*None or once in a while*	5691 (34.53)	356 (17.61)	
*1–2 times weekly*	3417 (20.73)	285 (14.09)	
*3–5 times weekly*	2540 (15.41)	412 (20.38)	
*Every day*	4833 (29.32)	969 (47.92)	
Health status			
*Food allergy, n (%)*	1658 (10.06)	239 (11.82)	0.014
*Drug allergy, n (%)*	694 (4.21)	81 (4.01)	0.664
*Dental caries, median (IQR)*	1 (0–2)	0 (0–2)	0.318
*Family history of diabetes, n (%)*	9925 (60.22)	1653 (81.75)	<0.001
Family information			
*Maternal reproductive age (years), median (IQR)*	1 (0–2)	1 (0–2)	<0.001
*Paternal reproductive age (years), median (IQR)*	1 (0–2)	1 (0–2)	
*Maternal BMI (kg/m^2^), median (IQR)*	27.41 (25.17–30.10)	28.61 (26.09–31.83)	<0.001
*Paternal BMI (kg/m^2^), median (IQR)*	28.41 (26.25–31.5)	29.92 (27.01–33.51)	<0.001
Maternal education level, n (%)			<0.001
*High school degree or below*	6677 (40.51)	689 (34.08)	
*Bachelor’s degree*	9039 (54.84)	1019 (50.40)	
*Master’s degree or above*	765 (4.64)	341 (15.53)	
Paternal education level, n (%)			<0.001
*High school degree or below*	7673 (46.56)	774 (38.28)	
*Bachelor’s degree*	7940 (48.18)	892 (44.11)	
*Master’s degree or above*	868 (5.27)	356 (17.61)	
Family income (RMB per year), n (%)			<0.001
*<100 000*	6964 (42.25)	731 (36.15)	
*100 000–300 000)*	7161 (43.35)	748 (36.11)	
*≥300 000*	2356 (14.4)	543 (27.74)	

BMI – body mass index, IQR – interquartile range, RMB – renminbi

*Malnutrition was defined according to the criteria recommended by the World Health Organization.

### Selection of optimal algorithm

**Figure 1:**
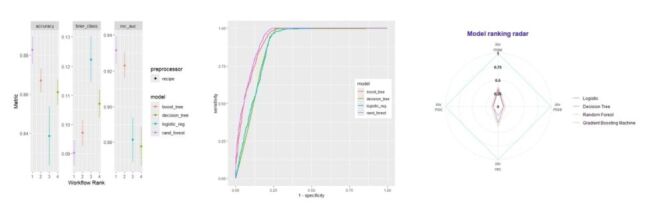
Predictive performance of four machine learning algorithms for child weight status. Malnutrition was defined using the criteria recommended by the World Health Organization. ROC – the receiver operating characteristic curve, GBM – Gradient Boosting Machine.

**Figure 1** illustrated the comparative performance of the Logistic regression model and three ensemble learning algorithms in the testing set. After thorough evaluation of AUROC for 4 predictive algorithms, random forest algorithm was teased out as the optimal algorithm, which had the highest AUROC score of 0.929, followed by GBM, decision tree, and Logistic regression. The superiority of the random forest algorithm over other algorithms was further reinforced by Brier score, MAE, MSE, REC, and RROC (**Figure 1**), as well as the visual DCA plot (Figure S1 in the **Online Supplementary Document**). After comprehensive evaluation, random forest was selected as the optimal algorithm in predicting child malnutrition.

### Determination of optimal important factors

**Figure 2:**
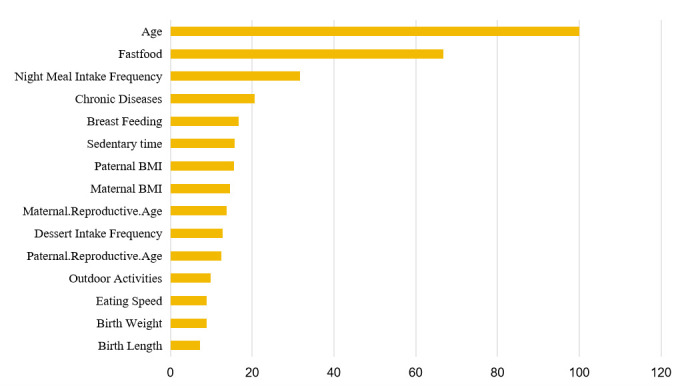
The ranking of 15 most important variables related to malnutrition based on the Random Forest model. Malnutrition was defined using the criteria recommended by the World Health Organization.

With the use of optimal random forest algorithm, **Table 2** showed the importance of the top 15 factors in the prediction of child malnutrition, and age, fast food intake frequency, night meal intake frequency, family history of diabetes, and breastfeeding time formed the top five important factors. With the increase in cumulating number of top factors, AUROC, accuracy, and prevision exhibited an increasing trend, from 0.891 (only age) to 0.924 (all top 15 important factors). By contrast, the changes in accuracy and precision were fluctuated, with the top 8 factors having the largest accuracy (0.880) and top 3 factors having the largest precision (0.969). The importance ranking of 15 top factors was displayed in **Figure 2**.

**Table 2:** Distributions of the area under the receiver operating curve, accuracy and precision with the cumulating number of top 15 factors in a descending order using the random forest algorithm for child malnutrition*

Variables	Cumulating number of top 15 factors	AUROC	Accuracy	Precision
Age	1	0.891	0.863	0.944
Fast food intake frequency	2	0.900	0.854	0.959
Night meal intake frequency	3	0.901	0.868	0.969
Family history of diabetes	4	0.902	0.874	0.958
Breastfeeding time	5	0.903	0.875	0.954
Sedentary time	6	0.905	0.871	0.952
Paternal BMI	7	0.916	0.868	0.939
Maternal BMI	8	0.925	0.880	0.956
Maternal reproductive age	9	0.922	0.879	0.946
Dessert intake frequency	10	0.922	0.878	0.949
Paternal reproductive age†	11	0.922	0.876	0.949
Outdoor activities	12	0.924	0.872	0.943
Eating speed	13	0.923	0.879	0.966
Birth weight	14	0.924	0.876	0.956
Birth length	15	0.924	0.877	0.964

AUROC – area under the receiver operating characteristic curve, BMI – body mass index

*Malnutrition status was defined using the criteria recommended by the World Health Organization.

†Subgroup thresholds for parental reproductive age are derived from restricted cubic spline curves.

### Risk quantification

Finally, Logistic regression was utilised to translate the clinical utilisation of the optimal set of important factors in practice. As shown in **Table 3**, at the 1‰ level, all factors were significantly associated with the risk of malnutrition in children and adolescents aged 3–14 years old. For example, in terms of fast food intake frequency, children who consumed fast foods 1–2 times weekly, 3–5 times weekly, and every day were 0.909 (95% CI = 0.755, 1.093, *P* = 0.309), 3.662 (95% CI = 3.120, 4.298, *P* < 0.001), and 4.028 (95% CI = 3.476, 4.668, *P* < 0.001) times more likely to be malnourished.

**Table 3:** The risk prediction of top eight factors for child malnutrition using Logistic regression model*

Top factors	OR† (95% CI)	*P*-value
Age		
*Pre-school children (3–6 y)*	Reference	
*School-age children (7–14 y)*	3.767 (3.984–4.180)	<0.001
Fast food intake frequency		
*None or once in a while*	Reference	
*1–2 times weekly*	0.909 (0.755–1.093)	0.309
*3–5 times weekly*	3.662 (3.120–4.298)	<0.001
*Every day*	4.028 (3.476–4.668)	<0.001
Night meal intake frequency		
*None or once in a while*	Reference	
*1–2 times weekly*	1.333 (1.135–1.567)	<0.001
*3–5 times weekly*	2.593 (2.233–3.011)	<0.001
*Every day*	3.205 (2.822–3.641)	<0.001
Family history of diabetes		
*No*	Reference	
*Yes*	2.959 (2.632–3.327)	<0.001
Breast feeding time		
*<6 mo*	0.601 (0.535–0.674)	<0.001
*6–24 mo*	Reference	
*≥24 mo*	1.360 (1.182–1.565)	<0.001
Sedentary time		
*<2 h per day*	Reference	
*≥2 h per day*	0.629 (0.569–0.694)	<0.001
Paternal BMI		
*Underweight (BMI<18.5 kg/m^2^)*	2.835 (2.253–3.597)	<0.001
*Normal (18.5 kg/m^2^≤BMI<25 kg/m^2^)*	Reference	
*Overweight or obesity (BMI≥25 kg/m^2^)*	0.758 (0.689–0.834)	<0.001
Maternal BMI		
*Underweight (BMI<18.5 kg/m^2^)*	1.580 (1.327–1.880)	<0.001
*Normal (18.5 kg/m^2^≤BMI<25 kg/m^2^)*	Reference	
*Overweight or obesity (BMI≥25 kg/m^2^)*	0.725 (0.649–0.809)	<0.001

BMI – body mass index, CI – confidence interval, OR – odds ratio

*Malnutrition was defined using the criteria recommended by the World Health Organization.

†ORs were adjusted for age, sex, education and family income.

### Convenient application for clinical utility

**Figure 3:**
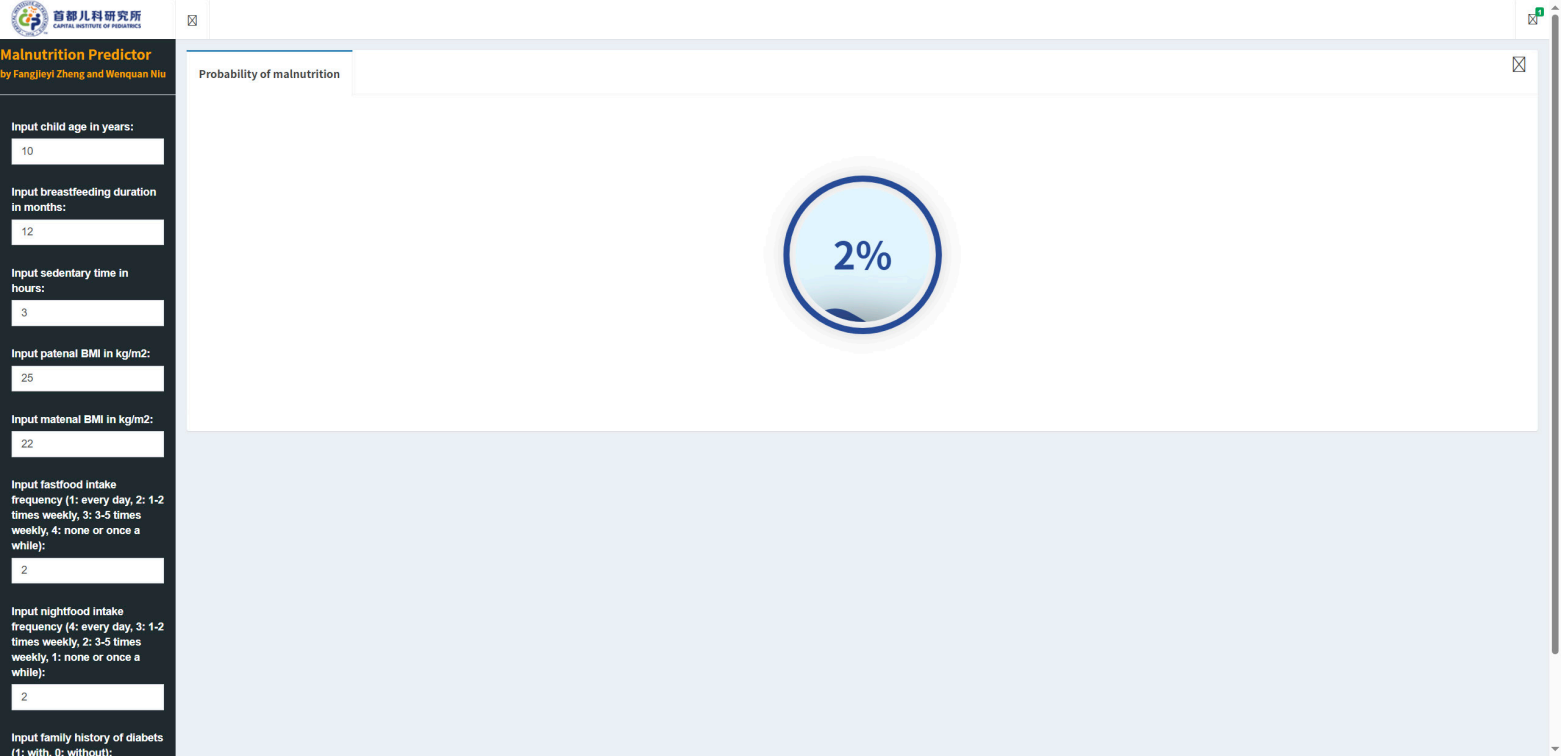
Convenient application for clinical utility. Weight status was defined according to the criteria recommended by the World Health Organization.

After comprehensive evaluation, the top 8 factors constituted the optimal set in predicting child malnutrition, including age, frequency of fast food intake, frequency of late-night snacking, family history of diabetes, duration of breastfeeding, sedentary time, and parents’ BMI. To facilitate practical calculation, an online child malnutrition assessment applet[18] was developed to help calculate the predicted probability of malnutrition in children aged 3–14 years (**Figure 3**).

## DISCUSSION

In this study, we aimed to estimate the prevalence of child malnutrition and determine a minimum set of culprit factors that can capture its risk by employing multiple machine learning algorithms among 18 503 children and adolescents 3–14 years from Beijing and Tangshan. After comprehensive evaluation and comparison, random forest stood out as the best algorithm in predicting child malnutrition, and it had the best predictive performance with an accuracy rate at 89%. Additionally, with the use of random forest algorithm, top 8 important factors proved to be sufficient to predict the risk of child malnutrition. To the best of our knowledge, this is thus far the first study to date that has utilised artificial intelligence technology to decipher the risk characteristics of malnutrition among preschool and school-age children in China.

Observational studies over the past decade have shown that overall detection rate of malnutrition among children across various regions of China has been on a downward trend [4,19]. As reflected in this study, over 10% of children aged 3–14 years were in a state of malnutrition, higher than that reported in 2019 among Chinese children aged 7–18 years at 8.64% [4]. The unusually elevated prevalence of malnutrition, indicative of both stunting and wasting among children, underscores the imperative nature of addressing this public health issue as a matter of urgency. Our prior research identified seven significant factors – parental education level, household income, frequency of fast food consumption, frequency of evening meal consumption, eating speed, maternal obesity, and parental obesity – as statistically significant predictors for child malnutrition [20]. With the emergence of artificial intelligence, our research methodologies have undergone optimisation, yielding more precise and comprehensive conclusions through enhanced data analyses and interpretation capabilities. We, in the present study, employed three ensemble learning algorithms and traditional Logistic regression, to explore which algorithm performed best, and to determine the minimal number of contributing factors that can sufficient in capability to predict child malnutrition.

After thoroughly comparing various performance indicators, including accuracy, Brier score, AUROC, DCA, MAE, MSE, REC, and RROC, we found that random forest consistently surpassed other algorithms in predicting child malnutrition. This finding is not surprising, as random forest has gained widespread acceptance in research due to its ability to enhance prediction accuracy by integrating a pre-determined number of decision trees. Furthermore, growing evidence suggests that random forest excels at managing complex, high-dimensional data sets with numerous factors, by either voting or averaging the predictions made by individual trees [21–25].

In this study, the random forest algorithm was utilised to ascertain the minimal number of contributing factors (n = 8) essential for predicting child malnutrition. Additionally, a comparative analysis was undertaken to evaluate the predictive performance of this algorithm when considering the full range of investigated factors, as opposed to the reduced set of eight causal factors. In contrast to the findings of our previous study, this study found that age was the primary factor influencing malnutrition, in line with the results of prior studies [26,27]. Given that age is an immutable factor, the present finding underscores the importance of enhanced vigilance among physicians and parents regarding the nutritional and developmental well-being of school-aged children.

Also, we found a strong correlation between poor dietary habits and malnutrition in children. Notably, the frequency of fast food and late-night snack consumption ranked second and third, respectively, among the most significant screened factors influencing malnutrition, consistent with findings by Khan et al. [28]. Moreover, our further Logistic regression analyses showed that the greater the frequency of fast food and late-night snacking, the greater the risk of malnutrition in children. Upon thorough analysis, we conclusively identified a familial history of diabetes as a noteworthy and moderate risk factor that predisposes children to malnutrition. One study found that chronic undernutrition has been linked to insulin deficiency and glucose intolerance [29]. Furthermore, our findings indicated that the duration of breastfeeding significantly impacted the incidence of child malnutrition. It is universally acknowledged that the recommended duration for exclusive breastfeeding spans from six to 24 months [30]. However, extending breastfeeding beyond this period may elevate the risk of having malnutrition among infants. Conversely, timely introduction of complementary foods within the first six months of lactation, alongside breastfeeding, notably mitigated the risk of malnutrition [31,32], similar to the results of our logistic regression analysis. Besides, echoing prior research [13,33], we observed a correlation between parental nutritional status and child malnutrition. Specifically, children of fathers and mothers with lower BMI exhibited a heightened prevalence of malnutrition. This linkage could stem from both inherited predispositions and lifestyle behaviours. Genetic factors have been demonstrated to play a role in the development of obesity and malnutrition, exerting their influence on metabolic processes, appetite regulation, and growth patterns. For instance, specific genetic disorders, such as leptin deficiency or melanocortin four receptor mutations, have been observed to predispose individuals to obesity [34]. Furthermore, genetic factors may influence how children respond to nutritional interventions in environments with poor sanitation and endemic diseases. The lifestyle behaviours of parents have been demonstrated to exert a significant influence on the dietary habits and levels of physical activity of their children. Families in which parents maintain a healthy BMI tend to exhibit a more conducive environment for healthy eating and exercise, which can mitigate the risk of obesity and malnutrition [34,35]. Conversely, parents with higher BMIs may model less healthy behaviours, thereby contributing to an obesogenic environment [34,36].

Some researchers have found that prolonged breastfeeding is associated with an increased risk of stunting and severe stunting in children aged 2–3 years, especially if it is not complemented by adequate solid foods [15]. This is because breast milk alone may not provide sufficient calories and nutrients for older children, leading to nutrient deficiencies if not balanced by appropriate complementary foods [37]. However, the relationship between breastfeeding duration and malnutrition may also be influenced by reverse causality, whereby mothers may continue breastfeeding longer due to suboptimal growth of their child [38].

For practical reasons, we integrated the minimal number of eight contributing factors into the random forest algorithm, and devised an online predictive tool to facilitate the calculation of child malnutrition, which can help inform clinical decisions and tailor prevention/intervention strategies for high-risk children.

### Limitations

Several limitations should be acknowledged in this study. First, all participants in this study were all from Beijing and Tangshan, and a note of caution should be sounded when applying our findings to the other regions and countries. Moreover, considering the cross-sectional nature of this study, causal links between factors of interest and child malnutrition cannot be addressed. Second, the causes of children’s underweight are complex; however, only 31 factors could be fed to machine learning algorithms. Further, the majority of included factors in our study were collected through self-reported questionnaires, leaving recall bias an open question. Additionally, machine learning algorithms demand substantial data to avoid overfitting and ensure model generalisability. The data was partitioned into a training set (70%) and a testing set (30%) to ensure sufficient statistical power. It is acknowledged that the absence of a formal sample size calculation may limit the interpretability of statistical power for specific associations. Nevertheless, the substantial sample size (n = 18 503) serves to reduce the risk of type II error and supports the reliability of the identified risk factors. In future studies, explicit sample size justifications will be prioritised to enhance methodological transparency. Third, only the WHO guideline for malnutrition was adopted in this study, and local guidelines were not used for the sake of comparability and generalisability. Last but not the least, because of the cross-sectional design, it is impossible to explore the causal relationship between potential risk factors and child malnutrition.

## CONCLUSIONS

Taken together, in 18 503 children and adolescents aged 3–14 years, out of three ensemble learning algorithms and traditional Logistic regression, we found that random forest stood out as the best algorithm in predicting child malnutrition, with an accuracy rate nearing 90%. Additionally, with the use of random forest algorithm, top 8 important factors proved to be sufficient to predict the risk of child malnutrition. Importantly, we have developed an online applet that formulates prediction based on our findings, which can enable the general public to utilise the model and facilitate the identification of children at high-risk for malnutrition, thereby enhance its clinical applicability.


Online Supplementary Document
