# Supplementary material for: Prevalence of malnutrition and associated factors in Chinese children and adolescents aged 3–14 years using machine learning algorithms
Source: J Glob Health. 2025 Jul 21;15:04204. doi: 10.7189/jogh.15.04204 (PMC12278689; doi:10.7189/jogh.15.04204)
Supplement: Online Supplementary Document [file jogh-15-04204-s001.pdf]

Supplement to: Fangjieyi Zheng, Kening Chen, Xiaoqian Zhang, Qiong Wang, Zhixin Zhang, Wenquan Niu. Prevalence of malnutrition and associated factors in Chinese children and adolescents aged 3–14 years using machine learning algorithms. J Glob Health. 2025;15:04204.

Figure S1. The DCA for predicting malnutrition using four different machine learning algorithms.

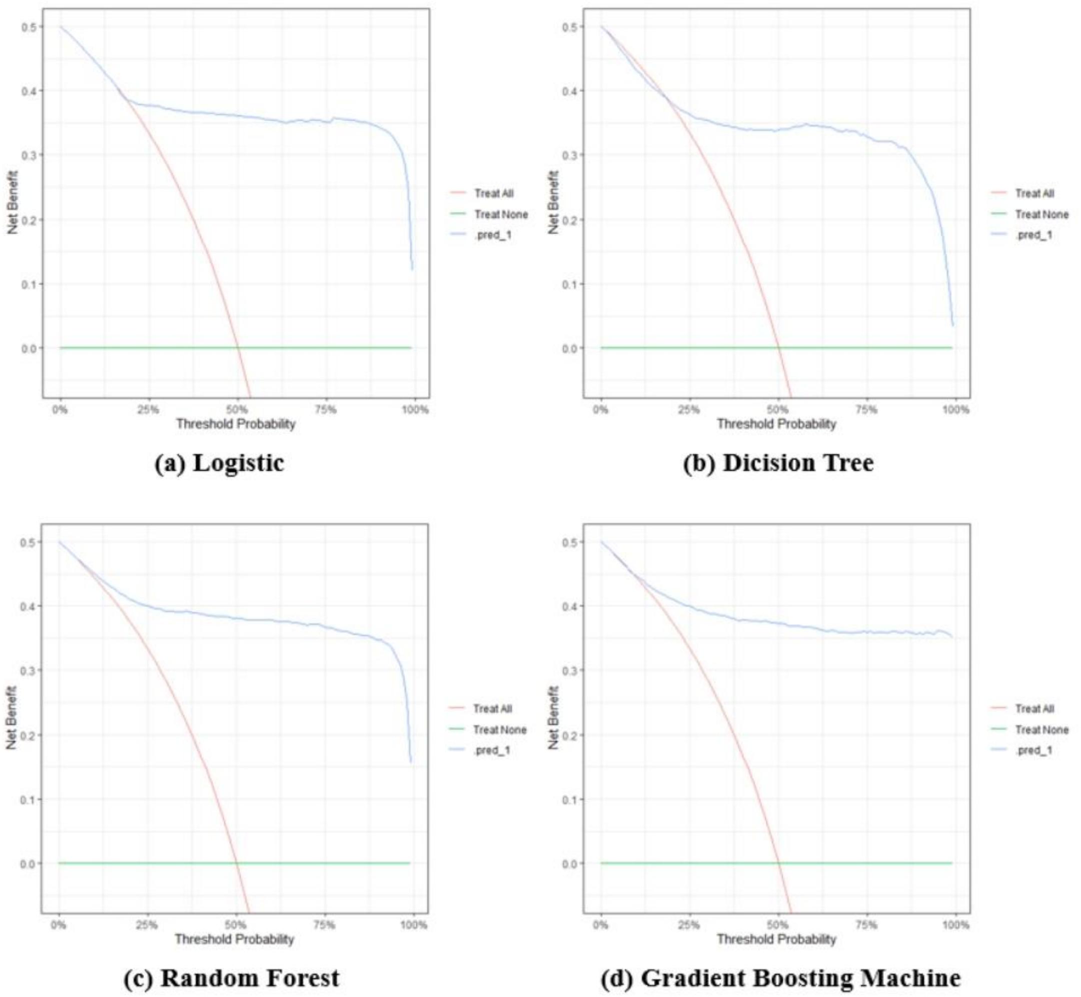

*Malnutrition status was defined using the criteria recommended by the World Health Organization (WHO).

Table S1. STROBE Statement—Checklist of items that should be included in reports of ***cross-sectional studies***

**Sections**	**Item No**	**Recommendation**	**Page No**
**Title and abstract**	1	(*a*) Indicate the study's design with a commonly used term in the title or the abstract	2
		(*b*) Provide in the abstract an informative and balanced summary of what was done and what was found	2-3
**Introduction**			
Background/rationale	2	Explain the scientific background and rationale for the investigation being reported	4
Objectives	3	State specific objectives, including any prespecified hypotheses	5
**Methods**			
Study design	4	Present key elements of study design early in the paper	5
Setting	5	Describe the setting, locations, and relevant dates, including periods of recruitment, exposure, follow-up, and data collection	7
Participants	6	(*a*) Give the eligibility criteria, and the sources and methods of selection of participants	5
Variables	7	Clearly define all outcomes, exposures, predictors, potential confounders, and effect modifiers. Give diagnostic criteria, if applicable	6
Data sources/ measurement	8*	For each variable of interest, give sources of data and details of methods of assessment (measurement). Describe comparability of assessment methods if there is more than one group	*6*
Bias	9	Describe any efforts to address potential sources of bias	10
Study size	10	Explain how the study size was arrived at	7
Quantitative variables	11	Explain how quantitative variables were handled in the analyses. If applicable, describe which groupings were chosen and why	7-8
Statistical methods	12	(*a*) Describe all statistical methods, including those used to control for confounding	9-10
		(*b*) Describe any methods used to examine subgroups and interactions	9-10
		(*c*) Explain how missing data were addressed	9
		(*d*) If applicable, describe analytical methods taking account of sampling strategy	9
		(*e*) Describe any sensitivity analyses	9
**Results**			
Participants	13*	(a) Report numbers of individuals at each stage of study—eg numbers potentially eligible, examined for eligibility, confirmed eligible, included in the study, completing follow-up, and analysed	6
		(b) Give reasons for non-participation at each stage	8
		(c) Consider use of a flow diagram	-
Descriptive data	14*	(a) Give characteristics of study participants (eg demographic, clinical, social) and information on exposures and potential confounders	11
		(b) Indicate number of participants with missing data for each variable of interest	7
Outcome data	15*	Report numbers of outcome events or summary measures	11
Main results	16	(*a*) Give unadjusted estimates and, if applicable, confounder-adjusted estimates and their precision (eg, 95% confidence interval). Make clear which confounders were adjusted for and why they were included	11
		(*b*) Report category boundaries when continuous variables were categorized	11
		(*c*) If relevant, consider translating estimates of relative risk into absolute risk for a meaningful time period	12

Other analyses	17	Report other analyses done—eg analyses of subgroups and interactions, and sensitivity analyses	12
**Discussion**			
Key results	18	Summarise key results with reference to study objectives	12-13
Limitations	19	Discuss limitations of the study, taking into account sources of potential bias or imprecision. Discuss both direction and magnitude of any potential bias	17-18
Interpretation	20	Give a cautious overall interpretation of results considering objectives, limitations, multiplicity of analyses, results from similar studies, and other relevant evidence	14-16
Generalisability	21	Discuss the generalisability (external validity) of the study results	18
**Other information**			
Funding	22	Give the source of funding and the role of the funders for the present study and, if applicable, for the original study on which the present article is based	18-19

*Give information separately for exposed and unexposed groups.

Note: An Explanation and Elaboration article discusses each checklist item and gives methodological background and published examples of transparent reporting. The STROBE checklist is best used in conjunction with this article (freely available on the Web sites of PLoS Medicine at http://www.plosmedicine.org/, Annals of Internal Medicine at http://www.annals.org/, and Epidemiology at http://www.epidem.com/). Information on the STROBE Initiative is available at www.strobe-statement.org.

File 1. R codes for the statistical performance of this study.

```
library(tidymodels)
library(bonsai)
library(parsnip)
library(discrim)
library(baguette)
library(MatchIt)
library(Matching)
library(tidyverse)
library(scales)

setwd("D:/")
df <- read.csv("jydata3.csv", header = TRUE, stringsAsFactors = FALSE)
dfs <- df %>%
  mutate_at(vars(-sex, -faeducation,  -maeducation, -foodallergy,  -drugallergy,  -uri, -lri,  -dentalcaries,  -
deliverymode, -bloodtype,  -assistedreproduction,  -pregnancyorder,  -deliveryorder,-twins,  -infancyfeeding,  -
fastfood,  -sweetfood,  -nightmeal,  -dm_kin, -asthma,  -rhinitis,  -eczema,  -ethnicity,   -income,  -malnutrition),
rescale)
dfs <- as.data.frame(dfs)
match.it <- matchit(malnutrition ~sex, data = dfs, method="nearest", ratio=1)
dfsm <- match.data(match.it)[1:ncol(dfs)]
dfsm$malnutrition <- as.factor(dfsm$malnutrition)
library(caTools)
set.seed(123)
split <- sample.split(dfsm$malnutrition, SplitRatio = 0.7)
traindata <- subset(dfsm, split == TRUE)
testdata <- subset(dfsm, split == FALSE)
log_spec <-
  logistic_reg() %>%
  set_engine('glm')
cart_spec <-
  decision_tree() %>%
  set_engine('rpart') %>%
  set_mode('classification')
randomforest_spec <-
  rand_forest() %>%
  set_engine('ranger') %>%
  set_mode('classification')
xgbm_spec <-
  boost_tree() %>%
  set_engine('xgboost') %>%
  set_mode('classification')
folds <- vfold_cv(traindata, v=5, strata = malnutrition)
ctr <- control_resamples(save_pred = TRUE)
rec <- recipe(malnutrition ~., data = traindata)
wf <- workflow_set(preproc = list(basic = rec),
                   models = list(log = log_spec,
```

```
                                        dtree = cart_spec,
                                        rf = randomforest_spec,
                                        xgbm = xgbm_spec))
wf_model <- wf %>%
  workflow_map("fit_resamples",
               resamples=folds,
               control=ctr)
#fn: The name of the function to run, as a character. Acceptable values are: "tune_grid", "tune_bayes", "fit_resamples", "tune_race_anova", "tune_race_win_loss", or "tune_sim_anneal". Note that users need not provide the namespace or parentheses in this argument, e.g. provide "tune_grid" rather than "tune::tune_grid" or "tune_grid()".
collect_predictions(wf_model) %>%
  conf_mat(malnutrition, .pred_class) %>%
  autoplot()
###AUC
autoplot(wf_model, metric = "roc_auc") +
  geom_text(aes(y = mean - 1/20, label = wflow_id),
            angle = 270,
            hjust = 1)+
  theme(legend.position = "none")+
  xlim(c(1, 4))
###Accuracy, Brier, AUC
library(tidymodels)
library(baguette)
library(discrim)
autoplot(wf_model)
###ROC
collect_predictions(wf_model) %>%
  group_by(model) %>%
  roc_curve(malnutrition, .pred_0) %>%
  ggplot(aes(x=1-specificity, y=sensitivity, color=model))+
  geom_line(lwd=0.8)+
  theme(legend.position = c(.98, .65),
        legend.justification = c("right", "top"))
###DCA
library(dcurves)
collect_predictions(wf_model) %>%
  group_by(wflow_id) %>%
  group_map(~dca(data = .x, malnutrition ~ .pred_1))
rf <- randomForest(as.factor(malnutrition)~., data = traindata, ntree=200, importance=TRUE)
predcont <- predict(rf, as.matrix(testdata[, c(-40)]), type="prob")
pred <- predict(rf, as.matrix(testdata[, c(-40)]))
rf$importance
testdata$Actual <- ifelse(testdata$malnutrition == 1, "Yes", "No")
testdata$Predicted <- ifelse(pred == 1, "Yes", "No")
confusionMatrix(as.factor(testdata$Predicted), as.factor(testdata$Actual), positive = "Yes")
CrossTable(testdata$malnutrition, pred, prop.chisq = F, prop.c = F, prop.r = F, dnn=c("Actual value", "Predicted value"))
dtree <- table(testdata$malnutrition, pred, dnn=c("Actual value", "Predicted value"))
```

```
performance <- function(table, n = 3) {
   if (!all(dim(table) == c(2, 2)))
      stop("Must be a 2*2 table")
   tn <- table[1,1]
   fp <- table[1,2]
   fn <- table[2,1]
   tp <- table[2,2]
   sensitivity <- tp/(tp+fn)
   specificity <- tn/(tn+fp)
   ppp <- tp/(tp+fp)
   npp <- tn/(tn+fn)
   hitrate <- (tp+tn)/(tp+tn+fp+fn)
   result <- paste("Sensitivity = ", round(sensitivity, n),
                   "\nSpecificity = ", round(specificity, n),
                   "\nPositive Predictive Value = ", round(ppp, n),
                   "\nNegative Predictive Value = ", round(npp, n),
                   "\nAccuracy = ", round(hitrate, n), "\n", sep="")
   cat(result)
}
performance(dtree)
roc <- pROC::roc(testdata$malnutrition, as.numeric(predcont[,2]))
plot(roc, main = "ROC Curve", print.auc = TRUE, auc.polygon = TRUE, grid = TRUE, legacy.axes = TRUE, col="blue")
setwd("D:/")
df <- read.csv("data3.csv", header = TRUE, stringsAsFactors = FALSE)
dfs <- df %>%
   mutate_at(vars(-sex, -faeducation,    -maeducation, -foodallergy,   -drugallergy,   -uri, -lri,  -dentalcaries,  -
deliverymode, -bloodtype,     -assistedreproduction,     -pregnancyorder,     -deliveryorder,-twins,     -infancyfeeding,     -
fastfood,  -sweetfood,     -nightmeal,     -dm_kin, -asthma,  -rhinitis,  -eczema,  -ethnicity,       -income,  -malnutrition),
rescale)
dfs <- as.data.frame(dfs)
match.it <- matchit(malnutrition ~sex, data = dfs, method="nearest", ratio=1)
dfsm <- match.data(match.it)[1:ncol(dfs)]
dfsm$malnutrition <- as.factor(dfsm$malnutrition)
set.seed(123)
split <- sample.split(dfsm$malnutrition, SplitRatio = 0.7)
traindata <- subset(dfsm, split == TRUE)
testdata <- subset(dfsm, split == FALSE)
rf <- randomForest(as.factor(malnutrition)~age_year , data = traindata, ntree=200, importance=TRUE)
predcont <- predict(rf, as.matrix(testdata[, c(-40)]), type="prob")
pred <- predict(rf, as.matrix(testdata[, c(-40)]))
rf$importance
testdata$Actual <- ifelse(testdata$malnutrition == 1, "Yes", "No")
testdata$Predicted <- ifelse(pred == 1, "Yes", "No")
confusionMatrix(as.factor(testdata$Predicted), as.factor(testdata$Actual), positive = "Yes")
CrossTable(testdata$malnutrition, pred, prop.chisq = F, prop.c = F, prop.r = F, dnn=c("Actual value", "Predicted value"))
dtree <- table(testdata$malnutrition, pred, dnn=c("Actual value", "Predicted value"))
performance <- function(table, n = 3) {
```

```
    if (!all(dim(table) == c(2, 2)))
        stop("Must be a 2*2 table")
    tn <- table[1,1]
    fp <- table[1,2]
    fn <- table[2,1]
    tp <- table[2,2]
    sensitivity <- tp/(tp+fn)
    specificity <- tn/(tn+fp)
    ppp <- tp/(tp+fp)
    npp <- tn/(tn+fn)
    hitrate <- (tp+tn)/(tp+tn+fp+fn)
    result <- paste("Sensitivity = ", round(sensitivity, n),
                    "\nSpecificity = ", round(specificity, n),
                    "\nPositive Predictive Value = ", round(ppp, n),
                    "\nNegative Predictive Value = ", round(npp, n),
                    "\nAccuracy = ", round(hitrate, n), "\n", sep="")
    cat(result)
}
performance(dtree)
roc <- pROC::roc(testdata$malnutrition, as.numeric(predcont[,2]))
plot(roc, main = "ROC Curve", print.auc = TRUE, auc.polygon = TRUE, grid = TRUE, legacy.axes = TRUE, col="blue")

setwd("D:/")
df <- read.csv("data3.csv", header = TRUE, stringsAsFactors = FALSE)
dfs <- df %>%
    mutate_at(vars(-sex, -faeducation, -maeducation, -foodallergy, -drugallergy, -uri, -lri, -dentalcaries, -deliverymode, -bloodtype, -assistedreproduction, -pregnancyorder, -deliveryorder,-twins, -infancyfeeding, -fastfood, -sweetfood, -nightmeal, -dm_kin, -asthma, -rhinitis, -eczema, -ethnicity, -income, -malnutrition), rescale)
dfs <- as.data.frame(dfs)
match.it <- matchit(malnutrition ~sex, data = dfs, method="nearest", ratio=1)
dfsm <- match.data(match.it)[1:ncol(dfs)]
dfsm$malnutrition <- as.factor(dfsm$malnutrition)
set.seed(123)
split <- sample.split(dfsm$malnutrition, SplitRatio = 0.7)
traindata <- subset(dfsm, split == TRUE)
testdata <- subset(dfsm, split == FALSE)
rf <- randomForest(as.factor(malnutrition)~age_year+fastfood , data = traindata, ntree=200, importance=TRUE)
predcont <- predict(rf, as.matrix(testdata[, c(-40)]), type="prob")
pred <- predict(rf, as.matrix(testdata[, c(-40)]))
rf$importance
testdata$Actual <- ifelse(testdata$malnutrition == 1, "Yes", "No")
testdata$Predicted <- ifelse(pred == 1, "Yes", "No")
confusionMatrix(as.factor(testdata$Predicted), as.factor(testdata$Actual), positive = "Yes")
CrossTable(testdata$malnutrition, pred, prop.chisq = F, prop.c = F, prop.r = F, dnn=c("Actual value", "Predicted value"))
dtree <- table(testdata$malnutrition, pred, dnn=c("Actual value", "Predicted value"))
performance <- function(table, n = 3) {
```

```
    if (!all(dim(table) == c(2, 2)))
        stop("Must be a 2*2 table")
    tn <- table[1,1]
    fp <- table[1,2]
    fn <- table[2,1]
    tp <- table[2,2]
    sensitivity <- tp/(tp+fn)
    specificity <- tn/(tn+fp)
    ppp <- tp/(tp+fp)
    npp <- tn/(tn+fn)
    hitrate <- (tp+tn)/(tp+tn+fp+fn)
    result <- paste("Sensitivity = ", round(sensitivity, n),
                    "\nSpecificity = ", round(specificity, n),
                    "\nPositive Predictive Value = ", round(ppp, n),
                    "\nNegative Predictive Value = ", round(npp, n),
                    "\nAccuracy = ", round(hitrate, n), "\n", sep="")
    cat(result)
}
performance(dtree)
roc <- pROC::roc(testdata$malnutrition, as.numeric(predcont[,2]))
plot(roc, main = "ROC Curve", print.auc = TRUE, auc.polygon = TRUE, grid = TRUE, legacy.axes = TRUE, col="blue")
setwd("D:/")
df <- read.csv("data3.csv", header = TRUE, stringsAsFactors = FALSE)
dfs <- df %>%
    mutate_at(vars(-sex, -faeducation,    -maeducation, -foodallergy,   -drugallergy,   -uri, -lri,  -dentalcaries,  -
deliverymode, -bloodtype,     -assistedreproduction,     -pregnancyorder,     -deliveryorder,-twins,     -infancyfeeding,     -
fastfood,  -sweetfood,     -nightmeal,     -dm_kin,  -asthma,  -rhinitis,  -eczema,  -ethnicity,        -income,  -malnutrition),
rescale)
dfs <- as.data.frame(dfs)
match.it <- matchit(malnutrition ~sex, data = dfs, method="nearest", ratio=1)
dfsm <- match.data(match.it)[1:ncol(dfs)]
dfsm$malnutrition <- as.factor(dfsm$malnutrition)
set.seed(123)
split <- sample.split(dfsm$malnutrition, SplitRatio = 0.7)
traindata <- subset(dfsm, split == TRUE)
testdata <- subset(dfsm, split == FALSE)
rf <- randomForest(as.factor(malnutrition)~age_year+fastfood+nightmeal    , data = traindata, ntree=200,
importance=TRUE)
predcont <- predict(rf, as.matrix(testdata[, c(-40)]), type="prob")
pred <- predict(rf, as.matrix(testdata[, c(-40)]))
rf$importance
testdata$Actual <- ifelse(testdata$malnutrition == 1, "Yes", "No")
testdata$Predicted <- ifelse(pred == 1, "Yes", "No")
confusionMatrix(as.factor(testdata$Predicted), as.factor(testdata$Actual), positive = "Yes")
CrossTable(testdata$malnutrition, pred, prop.chisq = F, prop.c = F, prop.r = F, dnn=c("Actual value", "Predicted value"))
dtree <- table(testdata$malnutrition, pred, dnn=c("Actual value", "Predicted value"))
performance <- function(table, n = 3) {
```

```
    if (!all(dim(table) == c(2, 2)))
        stop("Must be a 2*2 table")
    tn <- table[1,1]
    fp <- table[1,2]
    fn <- table[2,1]
    tp <- table[2,2]
    sensitivity <- tp/(tp+fn)
    specificity <- tn/(tn+fp)
    ppp <- tp/(tp+fp)
    npp <- tn/(tn+fn)
    hitrate <- (tp+tn)/(tp+tn+fp+fn)
    result <- paste("Sensitivity = ", round(sensitivity, n),
                              "\nSpecificity = ", round(specificity, n),
                              "\nPositive Predictive Value = ", round(ppp, n),
                              "\nNegative Predictive Value = ", round(npp, n),
                              "\nAccuracy = ", round(hitrate, n), "\n", sep="")
    cat(result)
}
performance(dtree)
roc <- pROC::roc(testdata$malnutrition, as.numeric(predcont[,2]))
plot(roc, main = "ROC Curve", print.auc = TRUE, auc.polygon = TRUE, grid = TRUE, legacy.axes = TRUE, col="blue")
setwd("D:/")
df <- read.csv("data3.csv", header = TRUE, stringsAsFactors = FALSE)
dfs <- df %>%
    mutate_at(vars(-sex, -faeducation,     -maeducation, -foodallergy,   -drugallergy,   -uri, -lri,  -dentalcaries,  -
deliverymode, -bloodtype,     -assistedreproduction,     -pregnancyorder,     -deliveryorder,-twins,     -infancyfeeding,     -
fastfood,  -sweetfood,     -nightmeal,     -dm_kin,  -asthma,   -rhinitis,   -eczema,  -ethnicity,        -income,  -malnutrition),
rescale)
dfs <- as.data.frame(dfs)
match.it <- matchit(malnutrition ~sex, data = dfs, method="nearest", ratio=1)
dfsm <- match.data(match.it)[1:ncol(dfs)]
dfsm$malnutrition <- as.factor(dfsm$malnutrition)
set.seed(123)
split <- sample.split(dfsm$malnutrition, SplitRatio = 0.7)
traindata <- subset(dfsm, split == TRUE)
testdata <- subset(dfsm, split == FALSE)
rf <- randomForest(as.factor(malnutrition)~age_year+fastfood+nightmeal +dm_kin, data = traindata, ntree=200,
importance=TRUE)
predcont <- predict(rf, as.matrix(testdata[, c(-40)]), type="prob")
pred <- predict(rf, as.matrix(testdata[, c(-40)]))
rf$importance
testdata$Actual <- ifelse(testdata$malnutrition == 1, "Yes", "No")
testdata$Predicted <- ifelse(pred == 1, "Yes", "No")
confusionMatrix(as.factor(testdata$Predicted), as.factor(testdata$Actual), positive = "Yes")
CrossTable(testdata$malnutrition, pred, prop.chisq = F, prop.c = F, prop.r = F, dnn=c("Actual value", "Predicted value"))
dtree <- table(testdata$malnutrition, pred, dnn=c("Actual value", "Predicted value"))
performance <- function(table, n = 3) {
```

```
    if (!all(dim(table) == c(2, 2)))
        stop("Must be a 2*2 table")
    tn <- table[1,1]
    fp <- table[1,2]
    fn <- table[2,1]
    tp <- table[2,2]
    sensitivity <- tp/(tp+fn)
    specificity <- tn/(tn+fp)
    ppp <- tp/(tp+fp)
    npp <- tn/(tn+fn)
    hitrate <- (tp+tn)/(tp+tn+fp+fn)
    result <- paste("Sensitivity = ", round(sensitivity, n),
                                "\nSpecificity = ", round(specificity, n),
                                "\nPositive Predictive Value = ", round(ppp, n),
                                "\nNegative Predictive Value = ", round(npp, n),
                                "\nAccuracy = ", round(hitrate, n), "\n", sep="")
    cat(result)
}
performance(dtree)
roc <- pROC::roc(testdata$malnutrition, as.numeric(predcont[,2]))
plot(roc, main = "ROC Curve", print.auc = TRUE, auc.polygon = TRUE, grid = TRUE, legacy.axes = TRUE, col="blue")
setwd("D:/")
df <- read.csv("data3.csv", header = TRUE, stringsAsFactors = FALSE)
dfs <- df %>%
    mutate_at(vars(-sex, -faeducation,    -maeducation, -foodallergy,   -drugallergy,   -uri, -lri,  -dentalcaries,  -
deliverymode, -bloodtype,    -assistedreproduction,    -pregnancyorder,    -deliveryorder,-twins,    -infancyfeeding,    -
fastfood,  -sweetfood,    -nightmeal,    -dm_kin,  -asthma,  -rhinitis,  -eczema,  -ethnicity,      -income,  -malnutrition),
rescale)
dfs <- as.data.frame(dfs)
match.it <- matchit(malnutrition ~sex, data = dfs, method="nearest", ratio=1)
dfsm <- match.data(match.it)[1:ncol(dfs)]
dfsm$malnutrition <- as.factor(dfsm$malnutrition)
set.seed(123)
split <- sample.split(dfsm$malnutrition, SplitRatio = 0.7)
traindata <- subset(dfsm, split == TRUE)
testdata <- subset(dfsm, split == FALSE)
rf <- randomForest(as.factor(malnutrition)~age_year+fastfood+nightmeal +dm_kin+breastfeeding , data = traindata,
ntree=200, importance=TRUE)
predcont <- predict(rf, as.matrix(testdata[, c(-40)]), type="prob")
pred <- predict(rf, as.matrix(testdata[, c(-40)]))
rf$importance
testdata$Actual <- ifelse(testdata$malnutrition == 1, "Yes", "No")
testdata$Predicted <- ifelse(pred == 1, "Yes", "No")
confusionMatrix(as.factor(testdata$Predicted), as.factor(testdata$Actual), positive = "Yes")
CrossTable(testdata$malnutrition, pred, prop.chisq = F, prop.c = F, prop.r = F, dnn=c("Actual value", "Predicted value"))
dtree <- table(testdata$malnutrition, pred, dnn=c("Actual value", "Predicted value"))
performance <- function(table, n = 3) {
```

```
  if (!all(dim(table) == c(2, 2)))
    stop("Must be a 2*2 table")
  tn <- table[1,1]
  fp <- table[1,2]
  fn <- table[2,1]
  tp <- table[2,2]
  sensitivity <- tp/(tp+fn)
  specificity <- tn/(tn+fp)
  ppp <- tp/(tp+fp)
  npp <- tn/(tn+fn)
  hitrate <- (tp+tn)/(tp+tn+fp+fn)
  result <- paste("Sensitivity = ", round(sensitivity, n),
                  "\nSpecificity = ", round(specificity, n),
                  "\nPositive Predictive Value = ", round(ppp, n),
                  "\nNegative Predictive Value = ", round(npp, n),
                  "\nAccuracy = ", round(hitrate, n), "\n", sep="")
  cat(result)
}
performance(dtree)
roc <- pROC::roc(testdata$malnutrition, as.numeric(predcont[,2]))
plot(roc, main = "ROC Curve", print.auc = TRUE, auc.polygon = TRUE, grid = TRUE, legacy.axes = TRUE, col="blue")
setwd("D:/")
df <- read.csv("data3.csv", header = TRUE, stringsAsFactors = FALSE)
dfs <- df %>%
  mutate_at(vars(-sex, -faeducation,  -maeducation, -foodallergy,  -drugallergy,  -uri, -lri,  -dentalcaries,  -
deliverymode, -bloodtype,   -assistedreproduction,   -pregnancyorder,   -deliveryorder,-twins,   -infancyfeeding,   -
fastfood,  -sweetfood,   -nightmeal,   -dm_kin, -asthma,  -rhinitis,  -eczema,  -ethnicity,    -income,  -malnutrition),
rescale)
dfs <- as.data.frame(dfs)
match.it <- matchit(malnutrition ~sex, data = dfs, method="nearest", ratio=1)
dfsm <- match.data(match.it)[1:ncol(dfs)]
dfsm$malnutrition <- as.factor(dfsm$malnutrition)
set.seed(123)
split <- sample.split(dfsm$malnutrition, SplitRatio = 0.7)
traindata <- subset(dfsm, split == TRUE)
testdata <- subset(dfsm, split == FALSE)
rf <- randomForest(as.factor(malnutrition)~age_year+fastfood+nightmeal +dm_kin+breastfeeding+sit , data = traindata,
ntree=200, importance=TRUE)
predcont <- predict(rf, as.matrix(testdata[, c(-40)]), type="prob")
pred <- predict(rf, as.matrix(testdata[, c(-40)]))
rf$importance
testdata$Actual <- ifelse(testdata$malnutrition == 1, "Yes", "No")
testdata$Predicted <- ifelse(pred == 1, "Yes", "No")
confusionMatrix(as.factor(testdata$Predicted), as.factor(testdata$Actual), positive = "Yes")
CrossTable(testdata$malnutrition, pred, prop.chisq = F, prop.c = F, prop.r = F, dnn=c("Actual value", "Predicted value"))
dtree <- table(testdata$malnutrition, pred, dnn=c("Actual value", "Predicted value"))
performance <- function(table, n = 3) {
```

```
    if (!all(dim(table) == c(2, 2)))
        stop("Must be a 2*2 table")
    tn <- table[1,1]
    fp <- table[1,2]
    fn <- table[2,1]
    tp <- table[2,2]
    sensitivity <- tp/(tp+fn)
    specificity <- tn/(tn+fp)
    ppp <- tp/(tp+fp)
    npp <- tn/(tn+fn)
    hitrate <- (tp+tn)/(tp+tn+fp+fn)
    result <- paste("Sensitivity = ", round(sensitivity, n),
                    "\nSpecificity = ", round(specificity, n),
                    "\nPositive Predictive Value = ", round(ppp, n),
                    "\nNegative Predictive Value = ", round(npp, n),
                    "\nAccuracy = ", round(hitrate, n), "\n", sep="")
    cat(result)
}
performance(dtree)
roc <- pROC::roc(testdata$malnutrition, as.numeric(predcont[,2]))
plot(roc, main = "ROC Curve", print.auc = TRUE, auc.polygon = TRUE, grid = TRUE, legacy.axes = TRUE, col="blue")

setwd("D:/")
df <- read.csv("data3.csv", header = TRUE, stringsAsFactors = FALSE)
dfs <- df %>%
    mutate_at(vars(-sex, -faeducation,     -maeducation, -foodallergy,   -drugallergy,   -uri, -lri,  -dentalcaries,  -
deliverymode, -bloodtype,     -assistedreproduction,     -pregnancyorder,     -deliveryorder,-twins,     -infancyfeeding,     -
fastfood,  -sweetfood,     -nightmeal,     -dm_kin, -asthma,  -rhinitis,  -eczema,  -ethnicity,       -income,  -malnutrition),
rescale)
dfs <- as.data.frame(dfs)
match.it <- matchit(malnutrition ~sex, data = dfs, method="nearest", ratio=1)
dfsm <- match.data(match.it)[1:ncol(dfs)]
dfsm$malnutrition <- as.factor(dfsm$malnutrition)
set.seed(123)
split <- sample.split(dfsm$malnutrition, SplitRatio = 0.7)
traindata <- subset(dfsm, split == TRUE)
testdata <- subset(dfsm, split == FALSE)
rf <- randomForest(as.factor(malnutrition)~age_year+fastfood+nightmeal +dm_kin+breastfeeding+sit , data = traindata,
ntree=200, importance=TRUE)
predcont <- predict(rf, as.matrix(testdata[, c(-40)]), type="prob")
pred <- predict(rf, as.matrix(testdata[, c(-40)]))
rf$importance
testdata$Actual <- ifelse(testdata$malnutrition == 1, "Yes", "No")
testdata$Predicted <- ifelse(pred == 1, "Yes", "No")
confusionMatrix(as.factor(testdata$Predicted), as.factor(testdata$Actual), positive = "Yes")
CrossTable(testdata$malnutrition, pred, prop.chisq = F, prop.c = F, prop.r = F, dnn=c("Actual value", "Predicted value"))
dtree <- table(testdata$malnutrition, pred, dnn=c("Actual value", "Predicted value"))
```

```
performance <- function(table, n = 3) {
    if (!all(dim(table) == c(2, 2)))
        stop("Must be a 2*2 table")
    tn <- table[1,1]
    fp <- table[1,2]
    fn <- table[2,1]
    tp <- table[2,2]
    sensitivity <- tp/(tp+fn)
    specificity <- tn/(tn+fp)
    ppp <- tp/(tp+fp)
    npp <- tn/(tn+fn)
    hitrate <- (tp+tn)/(tp+tn+fp+fn)
    result <- paste("Sensitivity = ", round(sensitivity, n),
                                "\nSpecificity = ", round(specificity, n),
                                "\nPositive Predictive Value = ", round(ppp, n),
                                "\nNegative Predictive Value = ", round(npp, n),
                                "\nAccuracy = ", round(hitrate, n), "\n", sep="")
    cat(result)
}
performance(dtree)
roc <- pROC::roc(testdata$malnutrition, as.numeric(predcont[,2]))
plot(roc, main = "ROC Curve", print.auc = TRUE, auc.polygon = TRUE, grid = TRUE, legacy.axes = TRUE, col="blue")

setwd("D:/")
df <- read.csv("data3.csv", header = TRUE, stringsAsFactors = FALSE)
dfs <- df %>%
    mutate_at(vars(-sex, -faeducation, -maeducation, -foodallergy, -drugallergy, -uri, -lri, -dentalcaries, -
deliverymode, -bloodtype, -assistedreproduction, -pregnancyorder, -deliveryorder,-twins, -infancyfeeding, -
fastfood, -sweetfood, -nightmeal, -dm_kin, -asthma, -rhinitis, -eczema, -ethnicity, -income, -malnutrition),
rescale)
dfs <- as.data.frame(dfs)
match.it <- matchit(malnutrition ~sex, data = dfs, method="nearest", ratio=1)
dfsm <- match.data(match.it)[1:ncol(dfs)]
dfsm$malnutrition <- as.factor(dfsm$malnutrition)
set.seed(123)
split <- sample.split(dfsm$malnutrition, SplitRatio = 0.7)
traindata <- subset(dfsm, split == TRUE)
testdata <- subset(dfsm, split == FALSE)
rf <- randomForest(as.factor(malnutrition)~age_year+fastfood+nightmeal +dm_kin+breastfeeding+sit+bmifa+bmima , data
= traindata, ntree=200, importance=TRUE)
predcont <- predict(rf, as.matrix(testdata[, c(-40)]), type="prob")
pred <- predict(rf, as.matrix(testdata[, c(-40)]))
rf$importance
testdata$Actual <- ifelse(testdata$malnutrition == 1, "Yes", "No")
testdata$Predicted <- ifelse(pred == 1, "Yes", "No")
confusionMatrix(as.factor(testdata$Predicted), as.factor(testdata$Actual), positive = "Yes")
```

```
CrossTable(testdata$malnutrition, pred, prop.chisq = F, prop.c = F, prop.r = F, dnn=c("Actual value", "Predicted value"))
dtree <- table(testdata$malnutrition, pred, dnn=c("Actual value", "Predicted value"))
performance <- function(table, n = 3) {
  if (!all(dim(table) == c(2, 2)))
    stop("Must be a 2*2 table")
  tn <- table[1,1]
  fp <- table[1,2]
  fn <- table[2,1]
  tp <- table[2,2]
  sensitivity <- tp/(tp+fn)
  specificity <- tn/(tn+fp)
  ppp <- tp/(tp+fp)
  npp <- tn/(tn+fn)
  hitrate <- (tp+tn)/(tp+tn+fp+fn)
  result <- paste("Sensitivity = ", round(sensitivity, n),
                  "\nSpecificity = ", round(specificity, n),
                  "\nPositive Predictive Value = ", round(ppp, n),
                  "\nNegative Predictive Value = ", round(npp, n),
                  "\nAccuracy = ", round(hitrate, n), "\n", sep="")
  cat(result)
}
performance(dtree)
roc <- pROC::roc(testdata$malnutrition, as.numeric(predcont[,2]))
plot(roc, main = "ROC Curve", print.auc = TRUE, auc.polygon = TRUE, grid = TRUE, legacy.axes = TRUE, col="blue")
setwd("D:/")
df <- read.csv("data3.csv", header = TRUE, stringsAsFactors = FALSE)
dfs <- df %>%
  mutate_at(vars(-sex, -faeducation, -maeducation, -foodallergy, -drugallergy, -uri, -lri, -dentalcaries, -
deliverymode, -bloodtype, -assistedreproduction, -pregnancyorder, -deliveryorder,-twins, -infancyfeeding, -
fastfood, -sweetfood, -nightmeal, -dm_kin, -asthma, -rhinitis, -eczema, -ethnicity, -income, -malnutrition),
rescale)
dfs <- as.data.frame(dfs)
match.it <- matchit(malnutrition ~sex, data = dfs, method="nearest", ratio=1)
dfsm <- match.data(match.it)[1:ncol(dfs)]
dfsm$malnutrition <- as.factor(dfsm$malnutrition)
set.seed(123)
split <- sample.split(dfsm$malnutrition, SplitRatio = 0.7)
traindata <- subset(dfsm, split == TRUE)
testdata <- subset(dfsm, split == FALSE)
rf <- randomForest(as.factor(malnutrition)~age_year+fastfood+nightmeal
+dm_kin+breastfeeding+sit+bmifa+bmima+Maternal.Reproductive.Age , data = traindata, ntree=200, importance=TRUE)
predcont <- predict(rf, as.matrix(testdata[, c(-40)]), type="prob")
pred <- predict(rf, as.matrix(testdata[, c(-40)]))
rf$importance
testdata$Actual <- ifelse(testdata$malnutrition == 1, "Yes", "No")
testdata$Predicted <- ifelse(pred == 1, "Yes", "No")
confusionMatrix(as.factor(testdata$Predicted), as.factor(testdata$Actual), positive = "Yes")
```

```
CrossTable(testdata$malnutrition, pred, prop.chisq = F, prop.c = F, prop.r = F, dnn=c("Actual value", "Predicted value"))
dtree <- table(testdata$malnutrition, pred, dnn=c("Actual value", "Predicted value"))
performance <- function(table, n = 3) {
  if (!all(dim(table) == c(2, 2)))
    stop("Must be a 2*2 table")
  tn <- table[1,1]
  fp <- table[1,2]
  fn <- table[2,1]
  tp <- table[2,2]
  sensitivity <- tp/(tp+fn)
  specificity <- tn/(tn+fp)
  ppp <- tp/(tp+fp)
  npp <- tn/(tn+fn)
  hitrate <- (tp+tn)/(tp+tn+fp+fn)
  result <- paste("Sensitivity = ", round(sensitivity, n),
                  "\nSpecificity = ", round(specificity, n),
                  "\nPositive Predictive Value = ", round(ppp, n),
                  "\nNegative Predictive Value = ", round(npp, n),
                  "\nAccuracy = ", round(hitrate, n), "\n", sep="")
  cat(result)
}
performance(dtree)
roc <- pROC::roc(testdata$malnutrition, as.numeric(predcont[,2]))
plot(roc, main = "ROC Curve", print.auc = TRUE, auc.polygon = TRUE, grid = TRUE, legacy.axes = TRUE, col="blue")
setwd("D:/")
df <- read.csv("data3.csv", header = TRUE, stringsAsFactors = FALSE)
dfs <- df %>%
  mutate_at(vars(-sex, -faeducation, -maeducation, -foodallergy, -drugallergy, -uri, -lri, -dentalcaries, -
deliverymode, -bloodtype, -assistedreproduction, -pregnancyorder, -deliveryorder,-twins, -infancyfeeding, -
fastfood, -sweetfood, -nightmeal, -dm_kin, -asthma, -rhinitis, -eczema, -ethnicity, -income, -malnutrition),
rescale)
dfs <- as.data.frame(dfs)
match.it <- matchit(malnutrition ~sex, data = dfs, method="nearest", ratio=1)
dfsm <- match.data(match.it)[1:ncol(dfs)]
dfsm$malnutrition <- as.factor(dfsm$malnutrition)
set.seed(123)
split <- sample.split(dfsm$malnutrition, SplitRatio = 0.7)
traindata <- subset(dfsm, split == TRUE)
testdata <- subset(dfsm, split == FALSE)
rf <- randomForest(as.factor(malnutrition)~age_year+fastfood+nightmeal
+dm_kin+breastfeeding+sit+bmifa+bmima+Maternal.Reproductive.Age+sweetfood, data = traindata, ntree=200,
importance=TRUE)
predcont <- predict(rf, as.matrix(testdata[, c(-40)]), type="prob")
pred <- predict(rf, as.matrix(testdata[, c(-40)]))
rf$importance
testdata$Actual <- ifelse(testdata$malnutrition == 1, "Yes", "No")
testdata$Predicted <- ifelse(pred == 1, "Yes", "No")
```

```
confusionMatrix(as.factor(testdata$Predicted), as.factor(testdata$Actual), positive = "Yes")
CrossTable(testdata$malnutrition, pred, prop.chisq = F, prop.c = F, prop.r = F, dnn=c("Actual value", "Predicted value"))
dtree <- table(testdata$malnutrition, pred, dnn=c("Actual value", "Predicted value"))
performance <- function(table, n = 3) {
  if (!all(dim(table) == c(2, 2)))
    stop("Must be a 2*2 table")
  tn <- table[1,1]
  fp <- table[1,2]
  fn <- table[2,1]
  tp <- table[2,2]
  sensitivity <- tp/(tp+fn)
  specificity <- tn/(tn+fp)
  ppp <- tp/(tp+fp)
  npp <- tn/(tn+fn)
  hitrate <- (tp+tn)/(tp+tn+fp+fn)
  result <- paste("Sensitivity = ", round(sensitivity, n),
                  "\nSpecificity = ", round(specificity, n),
                  "\nPositive Predictive Value = ", round(ppp, n),
                  "\nNegative Predictive Value = ", round(npp, n),
                  "\nAccuracy = ", round(hitrate, n), "\n", sep="")
  cat(result)
}
performance(dtree)
roc <- pROC::roc(testdata$malnutrition, as.numeric(predcont[,2]))
plot(roc, main = "ROC Curve", print.auc = TRUE, auc.polygon = TRUE, grid = TRUE, legacy.axes = TRUE, col="blue")
setwd("D:/")
df <- read.csv("data3.csv", header = TRUE, stringsAsFactors = FALSE)
dfs <- df %>%
  mutate_at(vars(-sex, -faeducation,    -maeducation, -foodallergy,   -drugallergy,   -uri, -lri,  -dentalcaries,  -
deliverymode, -bloodtype,     -assistedreproduction,     -pregnancyorder,     -deliveryorder,-twins,     -infancyfeeding,     -
fastfood,  -sweetfood,     -nightmeal,     -dm_kin, -asthma,  -rhinitis,  -eczema,  -ethnicity,       -income,  -malnutrition),
rescale)
dfs <- as.data.frame(dfs)
match.it <- matchit(malnutrition ~sex, data = dfs, method="nearest", ratio=1)
dfsm <- match.data(match.it)[1:ncol(dfs)]
dfsm$malnutrition <- as.factor(dfsm$malnutrition)
set.seed(123)
split <- sample.split(dfsm$malnutrition, SplitRatio = 0.7)
traindata <- subset(dfsm, split == TRUE)
testdata <- subset(dfsm, split == FALSE)
rf <- randomForest(as.factor(malnutrition)~age_year+fastfood+nightmeal
+dm_kin+breastfeeding+sit+bmifa+bmima+Maternal.Reproductive.Age+sweetfood +Paternal.Reproductive.Age+outdoor ,
data = traindata, ntree=200, importance=TRUE)
predcont <- predict(rf, as.matrix(testdata[, c(-40)]), type="prob")
pred <- predict(rf, as.matrix(testdata[, c(-40)]))
rf$importance
testdata$Actual <- ifelse(testdata$malnutrition == 1, "Yes", "No")
```

```
testdata$Predicted <- ifelse(pred == 1, "Yes", "No")
confusionMatrix(as.factor(testdata$Predicted), as.factor(testdata$Actual), positive = "Yes")
CrossTable(testdata$malnutrition, pred, prop.chisq = F, prop.c = F, prop.r = F, dnn=c("Actual value", "Predicted value"))
dtree <- table(testdata$malnutrition, pred, dnn=c("Actual value", "Predicted value"))
performance <- function(table, n = 3) {
  if (!all(dim(table) == c(2, 2)))
    stop("Must be a 2*2 table")
  tn <- table[1,1]
  fp <- table[1,2]
  fn <- table[2,1]
  tp <- table[2,2]
  sensitivity <- tp/(tp+fn)
  specificity <- tn/(tn+fp)
  ppp <- tp/(tp+fp)
  npp <- tn/(tn+fn)
  hitrate <- (tp+tn)/(tp+tn+fp+fn)
  result <- paste("Sensitivity = ", round(sensitivity, n),
                  "\nSpecificity = ", round(specificity, n),
                  "\nPositive Predictive Value = ", round(ppp, n),
                  "\nNegative Predictive Value = ", round(npp, n),
                  "\nAccuracy = ", round(hitrate, n), "\n", sep="")
  cat(result)
}
performance(dtree)
roc <- pROC::roc(testdata$malnutrition, as.numeric(predcont[,2]))
plot(roc, main = "ROC Curve", print.auc = TRUE, auc.polygon = TRUE, grid = TRUE, legacy.axes = TRUE, col="blue")
```

```
setwd("D:/")
df <- read.csv("data3.csv", header = TRUE, stringsAsFactors = FALSE)
dfs <- df %>%
  mutate_at(vars(-sex, -faeducation,  -maeducation, -foodallergy,  -drugallergy,  -uri, -lri,  -dentalcaries,  -
deliverymode, -bloodtype,   -assistedreproduction,   -pregnancyorder,   -deliveryorder,-twins,   -infancyfeeding,   -
fastfood,  -sweetfood,   -nightmeal,   -dm_kin, -asthma,  -rhinitis,  -eczema,  -ethnicity,    -income,  -malnutrition),
rescale)
dfs <- as.data.frame(dfs)
match.it <- matchit(malnutrition ~sex, data = dfs, method="nearest", ratio=1)
dfsm <- match.data(match.it)[1:ncol(dfs)]
dfsm$malnutrition <- as.factor(dfsm$malnutrition)
set.seed(123)
split <- sample.split(dfsm$malnutrition, SplitRatio = 0.7)
traindata <- subset(dfsm, split == TRUE)
testdata <- subset(dfsm, split == FALSE)
rf <- randomForest(as.factor(malnutrition)~age_year+fastfood+nightmeal
+dm_kin+breastfeeding+sit+bmifa+bmima+Maternal.Reproductive.Age+sweetfood +Paternal.Reproductive.Age , data =
traindata, ntree=200, importance=TRUE)
predcont <- predict(rf, as.matrix(testdata[, c(-40)]), type="prob")
pred <- predict(rf, as.matrix(testdata[, c(-40)]))
```

```
rf$importance
testdata$Actual <- ifelse(testdata$malnutrition == 1, "Yes", "No")
testdata$Predicted <- ifelse(pred == 1, "Yes", "No")
confusionMatrix(as.factor(testdata$Predicted), as.factor(testdata$Actual), positive = "Yes")
CrossTable(testdata$malnutrition, pred, prop.chisq = F, prop.c = F, prop.r = F, dnn=c("Actual value", "Predicted value"))
dtree <- table(testdata$malnutrition, pred, dnn=c("Actual value", "Predicted value"))
performance <- function(table, n = 3) {
  if (!all(dim(table) == c(2, 2)))
    stop("Must be a 2*2 table")
  tn <- table[1,1]
  fp <- table[1,2]
  fn <- table[2,1]
  tp <- table[2,2]
  sensitivity <- tp/(tp+fn)
  specificity <- tn/(tn+fp)
  ppp <- tp/(tp+fp)
  npp <- tn/(tn+fn)
  hitrate <- (tp+tn)/(tp+tn+fp+fn)
  result <- paste("Sensitivity = ", round(sensitivity, n),
                  "\nSpecificity = ", round(specificity, n),
                  "\nPositive Predictive Value = ", round(ppp, n),
                  "\nNegative Predictive Value = ", round(npp, n),
                  "\nAccuracy = ", round(hitrate, n), "\n", sep="")
  cat(result)
}
performance(dtree)
roc <- pROC::roc(testdata$malnutrition, as.numeric(predcont[,2]))
plot(roc, main = "ROC Curve", print.auc = TRUE, auc.polygon = TRUE, grid = TRUE, legacy.axes = TRUE, col="blue")
setwd("D:/")
df <- read.csv("data3.csv", header = TRUE, stringsAsFactors = FALSE)
dfs <- df %>%
  mutate_at(vars(-sex, -faeducation,    -maeducation, -foodallergy,   -drugallergy,   -uri, -lri,  -dentalcaries,  -
deliverymode, -bloodtype,     -assistedreproduction,     -pregnancyorder,     -deliveryorder,-twins,     -infancyfeeding,     -
fastfood,  -sweetfood,     -nightmeal,     -dm_kin,  -asthma,  -rhinitis,  -eczema,  -ethnicity,       -income,  -malnutrition),
rescale)
dfs <- as.data.frame(dfs)
match.it <- matchit(malnutrition ~sex, data = dfs, method="nearest", ratio=1)
dfsm <- match.data(match.it)[1:ncol(dfs)]
dfsm$malnutrition <- as.factor(dfsm$malnutrition)
set.seed(123)
split <- sample.split(dfsm$malnutrition, SplitRatio = 0.7)
traindata <- subset(dfsm, split == TRUE)
testdata <- subset(dfsm, split == FALSE)
rf <- randomForest(as.factor(malnutrition)~age_year+fastfood+nightmeal
+dm_kin+breastfeeding+sit+bmifa+bmima+Maternal.Reproductive.Age+sweetfood +Paternal.Reproductive.Age+outdoor
+eatspeed, data = traindata, ntree=200, importance=TRUE)
predcont <- predict(rf, as.matrix(testdata[, c(-40)]), type="prob")
```

```
pred <- predict(rf, as.matrix(testdata[, c(-40)]))
rf$importance
testdata$Actual <- ifelse(testdata$malnutrition == 1, "Yes", "No")
testdata$Predicted <- ifelse(pred == 1, "Yes", "No")
confusionMatrix(as.factor(testdata$Predicted), as.factor(testdata$Actual), positive = "Yes")
CrossTable(testdata$malnutrition, pred, prop.chisq = F, prop.c = F, prop.r = F, dnn=c("Actual value", "Predicted value"))
dtree <- table(testdata$malnutrition, pred, dnn=c("Actual value", "Predicted value"))
performance <- function(table, n = 3) {
  if (!all(dim(table) == c(2, 2)))
    stop("Must be a 2*2 table")
  tn <- table[1,1]
  fp <- table[1,2]
  fn <- table[2,1]
  tp <- table[2,2]
  sensitivity <- tp/(tp+fn)
  specificity <- tn/(tn+fp)
  ppp <- tp/(tp+fp)
  npp <- tn/(tn+fn)
  hitrate <- (tp+tn)/(tp+tn+fp+fn)
  result <- paste("Sensitivity = ", round(sensitivity, n),
                  "\nSpecificity = ", round(specificity, n),
                  "\nPositive Predictive Value = ", round(ppp, n),
                  "\nNegative Predictive Value = ", round(npp, n),
                  "\nAccuracy = ", round(hitrate, n), "\n", sep="")
  cat(result)
}
performance(dtree)
roc <- pROC::roc(testdata$malnutrition, as.numeric(predcont[,2]))
plot(roc, main = "ROC Curve", print.auc = TRUE, auc.polygon = TRUE, grid = TRUE, legacy.axes = TRUE, col="blue")

setwd("D:/")
df <- read.csv("data3.csv", header = TRUE, stringsAsFactors = FALSE)
dfs <- df %>%
  mutate_at(vars(-sex, -faeducation, -maeducation, -foodallergy, -drugallergy, -uri, -lri, -dentalcaries, -deliverymode, -bloodtype, -assistedreproduction, -pregnancyorder, -deliveryorder,-twins, -infancyfeeding, -fastfood, -sweetfood, -nightmeal, -dm_kin, -asthma, -rhinitis, -eczema, -ethnicity, -income, -malnutrition), rescale)
dfs <- as.data.frame(dfs)
match.it <- matchit(malnutrition ~sex, data = dfs, method="nearest", ratio=1)
dfsm <- match.data(match.it)[1:ncol(dfs)]
dfsm$malnutrition <- as.factor(dfsm$malnutrition)
set.seed(123)
split <- sample.split(dfsm$malnutrition, SplitRatio = 0.7)
traindata <- subset(dfsm, split == TRUE)
testdata <- subset(dfsm, split == FALSE)
```

```
rf <- randomForest(as.factor(malnutrition)~age_year+fastfood+nightmeal
+dm_kin+breastfeeding+sit+bmifa+bmima+Maternal.Reproductive.Age+sweetfood +Paternal.Reproductive.Age+outdoor
+eatspeed+birthheight, data = traindata, ntree=200, importance=TRUE)
predcont <- predict(rf, as.matrix(testdata[, c(-40)]), type="prob")
pred <- predict(rf, as.matrix(testdata[, c(-40)]))
rf$importance
testdata$Actual <- ifelse(testdata$malnutrition == 1, "Yes", "No")
testdata$Predicted <- ifelse(pred == 1, "Yes", "No")
confusionMatrix(as.factor(testdata$Predicted), as.factor(testdata$Actual), positive = "Yes")
CrossTable(testdata$malnutrition, pred, prop.chisq = F, prop.c = F, prop.r = F, dnn=c("Actual value", "Predicted value"))
dtree <- table(testdata$malnutrition, pred, dnn=c("Actual value", "Predicted value"))
performance <- function(table, n = 3) {
  if (!all(dim(table) == c(2, 2)))
    stop("Must be a 2*2 table")
  tn <- table[1,1]
  fp <- table[1,2]
  fn <- table[2,1]
  tp <- table[2,2]
  sensitivity <- tp/(tp+fn)
  specificity <- tn/(tn+fp)
  ppp <- tp/(tp+fp)
  npp <- tn/(tn+fn)
  hitrate <- (tp+tn)/(tp+tn+fp+fn)
  result <- paste("Sensitivity = ", round(sensitivity, n),
                  "\nSpecificity = ", round(specificity, n),
                  "\nPositive Predictive Value = ", round(ppp, n),
                  "\nNegative Predictive Value = ", round(npp, n),
                  "\nAccuracy = ", round(hitrate, n), "\n", sep="")
  cat(result)
}
performance(dtree)
roc <- pROC::roc(testdata$malnutrition, as.numeric(predcont[,2]))
plot(roc, main = "ROC Curve", print.auc = TRUE, auc.polygon = TRUE, grid = TRUE, legacy.axes = TRUE, col="blue")

setwd("D:/")
df <- read.csv("data3.csv", header = TRUE, stringsAsFactors = FALSE)
dfs <- df %>%
  mutate_at(vars(-sex, -faeducation,   -maeducation, -foodallergy,  -drugallergy,  -uri, -lri,  -dentalcaries,  -
deliverymode, -bloodtype,   -assistedreproduction,   -pregnancyorder,   -deliveryorder,-twins,   -infancyfeeding,   -
fastfood,  -sweetfood,   -nightmeal,   -dm_kin, -asthma,  -rhinitis,  -eczema,  -ethnicity,    -income,  -malnutrition),
rescale)
dfs <- as.data.frame(dfs)
match.it <- matchit(malnutrition ~sex, data = dfs, method="nearest", ratio=1)
dfsm <- match.data(match.it)[1:ncol(dfs)]
dfsm$malnutrition <- as.factor(dfsm$malnutrition)
set.seed(123)
split <- sample.split(dfsm$malnutrition, SplitRatio = 0.7)
```

```
traindata <- subset(dfsm, split == TRUE)
testdata <- subset(dfsm, split == FALSE)
rf <- randomForest(as.factor(malnutrition)~age_year+fastfood+nightmeal
+dm_kin+breastfeeding+sit+bmifa+bmima+Maternal.Reproductive.Age+sweetfood +Paternal.Reproductive.Age+outdoor
+eatspeed+birthweight+birthheight, data = traindata, ntree=200, importance=TRUE)
predcont <- predict(rf, as.matrix(testdata[, c(-40)]), type="prob")
pred <- predict(rf, as.matrix(testdata[, c(-40)]))
rf$importance
testdata$Actual <- ifelse(testdata$malnutrition == 1, "Yes", "No")
testdata$Predicted <- ifelse(pred == 1, "Yes", "No")
confusionMatrix(as.factor(testdata$Predicted), as.factor(testdata$Actual), positive = "Yes")
CrossTable(testdata$malnutrition, pred, prop.chisq = F, prop.c = F, prop.r = F, dnn=c("Actual value", "Predicted value"))
dtree <- table(testdata$malnutrition, pred, dnn=c("Actual value", "Predicted value"))
performance <- function(table, n = 3) {
  if (!all(dim(table) == c(2, 2)))
    stop("Must be a 2*2 table")
  tn <- table[1,1]
  fp <- table[1,2]
  fn <- table[2,1]
  tp <- table[2,2]
  sensitivity <- tp/(tp+fn)
  specificity <- tn/(tn+fp)
  ppp <- tp/(tp+fp)
  npp <- tn/(tn+fn)
  hitrate <- (tp+tn)/(tp+tn+fp+fn)
  result <- paste("Sensitivity = ", round(sensitivity, n),
                  "\nSpecificity = ", round(specificity, n),
                  "\nPositive Predictive Value = ", round(ppp, n),
                  "\nNegative Predictive Value = ", round(npp, n),
                  "\nAccuracy = ", round(hitrate, n), "\n", sep="")
  cat(result)
}
performance(dtree)
roc <- pROC::roc(testdata$malnutrition, as.numeric(predcont[,2]))
plot(roc, main = "ROC Curve", print.auc = TRUE, auc.polygon = TRUE, grid = TRUE, legacy.axes = TRUE, col="blue")
```
